# On the Mercurial Action in Syphilis

**Published:** 1825-03

**Authors:** Thomas Pollock


### Art. II.—*On the Mercurial Action in Syphilis.*

By Thomas Pollock, Esq.

Why are the preparations of mercury, remedies in syphilis?—Because, apparently, they are the means of conveying oxygen into the system. The probability that this is their mode of action, may be rendered manifest when the three following facts are assented to.

1st. That, in syphilis, there is a tendency in the solids to change their form, to produce an eruption, a scurf, ulceration in the tonsils, uvula, palate, and a propensity in the bones to caries: in health, these symptoms are absent. 2d. Nitric acid has sometimes succeeded in curing syphilis. 3d. A person under the influence of mercury is more susceptible of the electric shock, than a person not so influenced.

Whenever the animal solids change form, ammonia appears to be in action; and, as in syphilis the solids have a greater propensity to change form than when in health, it may be satisfactory to inquire what do the solids produce when their form is changed by heat.

Spirit of hartshorn (liquor volatilis cornu cervi) may be procured from all animal matter, except the fat; and from bones it is generally procured by exposing them to distillation: the result is, carbonate of ammonia, empyreumatic oil, carburetted hydrogen, often containing sulphur and phosphorus, carbonic acid, and water. These are the product of the gelatine of the bones, and the gelatinous and cartilaginous parts of the body appear to be more particularly the seat of the morbid change going on in syphilis; and the residue remaining in the still is the calcined hartshorn, which consists almost entirely of the phosphate of lime. It is not here to be understood that the change the gelatine undergoes in this process, is precisely analogous to that it undergoes in caries in syphilis; but certain it is that the gelatine which, in the above process, yields ammonia, becomes, in caries, removed or changed in form, and most probably then likewise forms ammonia.

Nitric acid, as a remedy in syphilis, has been found of great service, alleviating the symptoms, and in some instances apparently removing the complaint; but, unlike the preparations of mercury, is not to be depended upon, as it has not in all cases prevented a return of the complaint, or what are generally termed secondary symptoms.

By considering in what circumstances the action of the oxides of mercury would differ from that of the nitric acid upon the above-mentioned substances, resulting from the decomposition of gelatine by heat; and by considering those properties in which mercury, as a metal, differs from other metals; and by considering in what circumstances that part of the surface of the body (the mouth), where the mercurial action is most clearly manifested, differs from other parts of the surface, it will appear that the above proposed theory of the mercurial action comes tolerably near the fact.

The action of the nitric acid upon the above-mentioned substances would be, that it would combine with the ammonia, forming a permanent compound, which would retard the formation of more ammonia, in the same manner as common salt, an analogous compound, would prevent animal matter undergoing the putrefactive process, thus far would arrest the progress of the disease; but this compound would probably not remain long in the situation where it was formed, but would be removed by the processes of the circulation and absorption constantly going on in the body.

But the action of the oxides of mercury upon the above resulting substances, would probably be very different from that of the nitric acid. If they would unite at all with the ammonia, the combination could not be permanent; but they probably would impart oxygen to the sulphur or phosphorus, and by their union with oxygen would be formed a substance possessing the properties of an acid, more especially that of forming a permanent compound with ammonia; while the metal, thus reduced to the metallic state, would probably be carried off into the circulating system, and, when it would come in contact with the atmosphere, would combine again with oxygen; and the oxide formed would again be absorbed and decomposed, by coming again in contact with that part where the morbid change was going on; so it is probable that the same mercury might, by these repeated processes of combination and decomposition, produce effect upon ammonia repeatedly, which nitric acid would only do once.

Mercury, as a metal, differs from all others, in being a specific in syphilis, a property no other is known to possess; and in having the degree of temperature at which it combines with oxygen, and that at which it is reduced to the metallic state, much nearer each other than those of any other metal; and upon the possession of these two properties so near, which in other metals are so distant from each other, the mercurial action appears to depend.

The mouth is the part where the influence of the mercury is the most perceptible, producing a metallic, copperish taste, and an increased flow of saliva: hence the term salivation, applied to the mercurial action. These circumstances appear to demonstrate the proposed theory,—at least as far as respects the agency of the atmosphere upon the mercury; because, owing to the function of breathing perpetually going on, fresh portions of atmospheric air must be constantly presented to the surfaces of the mouth and salival glands; and likewise, by the constant flow of saliva, any matter, as the mercury, supposed to exist in it, must be freely exposed to the action of the atmosphere.

Thus from the above it will appear that nitric acid, and the oxides of mercury, act as remedies in syphilis, owing to their powers, directly or indirectly, of neutralising the properties of ammonia, and thus resisting change in animal matter; and that the oxides are preferable, as their action is longer continued.

Here, perhaps, the inquiry may be put—do antiseptics owe their power to this property of neutralising ammonia? Yes. Witness all acids, some metallic oxides, and some other substances, which possess the power of combining with ammonia. Perhaps, a farther inquiry may be put—why should these compounds of ammonia, when formed, resist change in animal matter? In reply, why does common salt possess this property? Between common salt and the saline compounds of ammonia, there is a strong analogy; and it appears probable that they act as antiseptics, because, being saturated with ammonia, that substance produces no further action upon them, and therefore its further formation will either cease or be retarded.

*East Smithfield; October 25th*, 1824.

